# Prevalence of atopy and allergic rhinitis in patients with adult non-cystic fibrosis bronchiectasis

**DOI:** 10.3906/sag-1807-229

**Published:** 2019-04-18

**Authors:** Elif Yelda NİKSARLIOĞLU, Rana IŞIK, Mehmet Atilla UYSAL, Derya ÜNAL, Güngör ÇAMSARI

**Affiliations:** 1 Department of Chest Disease, University of Health Sciences, Yedikule Chest Diseases and Thoracic Surgery Training andResearch Hospital, İstanbul Turkey; 2 Department of Adult Allergy Unit, University of Health Sciences, Yedikule Chest Diseases and Thoracic Surgery Training and Research Hospital, İstanbul Turkey

**Keywords:** Allergic rhinitis, atopy, bronchiectasis, prevalence, skin prick test, symptoms

## Abstract

**Background/aim:**

Non-cystic fibrosis bronchiectasis (non-CF BR) is common in developing countries.****Limited data are available regarding the impact of atopy, and no data are available regarding allergic rhinitis in patients with adult bronchiectasis.****The aim of this study was to evaluate the prevalence of atopy and allergic rhinitis in the clinical conditions of patients with BR.

**Materials and methods:**

The study enrolled 101 patients who were diagnosed with non-CF BR using high-resolution computed chest tomography. Allergic rhinitis (AR) was defined by skin prick test (SPT) positivity and the presence of any nasal symptoms (watery runny nose, nasal obstruction, nasal itching, and sneezing).

**Results:**

The mean age of patients was 48 ± 15 years (range 18–82); 55 (54.5%) patients were female. SPT positivity was detected in 37 (36.6%) cases. AR was detected in 32 (31.7%) patients with non-CF BR. AR was related to dyspnea (P = 0.04) and number of admissions to an emergency department in the previous year (P = 0.01). Forced expiratory volume in 1 s and forced vital capacity in patients with and without AR were different (P = 0.01 and P = 0.01, respectively). AR was correlated with number of admissions to an emergency department in the last year (r = 0.417, P = 0.005).

**Conclusion:**

We concluded that atopy was detected in more than one-third of adult non-CF BR patients. This study demonstrated that non-CF BR patients might have AR; it might be important to be aware of nasal symptoms in non-CF BR patients.

## 1. Introduction

Non-cystic fibrosis bronchiectasis (non-CF BR) is characterized by irreversible destruction and dilatation of bronchi and bronchioles associated with frequent bacterial infections and inflammation (1). The most common symptoms include chronic cough, production of mucoid or purulent sputum, dyspnea, and intermittent hemoptysis (2). The aims of BR treatment are to control symptoms, maintain or improve pulmonary function, reduce exacerbation, and improve quality of life (2). Although BR is most commonly due to cystic fibrosis in developed countries, non-CF BR is an important condition in developing countries.

Atopy is defined by skin prick test (SPT) positivity to aeroallergens and/or elevations in total/specific IgE levels (3). In 1989, Pang et al. evaluated SPT results and obtained measurements of bronchial hyperreactivity of histamine and methacholine in 36 stable BR patients. Nine (25%) patients had positive SPT results with BR (4). Ozturk et al. reported that atopy was more frequent in patients with BR (48%) compared with healthy subjects (5).

Allergic rhinitis (AR) is the most common form of allergic disorder worldwide that significantly impacts patients’ quality of life (6). Accordini et al. reported that the risk of hospitalization and the number of days with impaired activity increased in adult AR patients (7). Some studies evaluated chronic rhinosinusitis (CRS) or sinonasal disease in BR patients (8,9). Guilemany et al. investigated the association of idiopathic and postinfective BR with CRS and nasal polyposis (NP) (9). They observed a high prevalence of CRS (77%) and NP (26%), and the severity of BR was related to the presence of CRS and NP. 

To our knowledge, limited studies investigating atopy are available, and data on AR in adult patients with BR are not available. The aim of this study was to evaluate the prevalence of atopy and AR in the clinical conditions of patients with BR.

## 2. Materials and methods

Two hundred and one patients with BR were evaluated in an outpatient clinic in our hospital between 2015 and 2017. One hundred patients were excluded due to allergic bronchopulmonary aspergillosis, cryoglobulinemic vasculitis, cystic fibrosis, interstitial lung diseases, BR exacerbation, or lack of data (Figure). An acute exacerbation was defined as having at least 3 of the following criteria: worsened dyspnea and coughing, an increase in the amount and purulence of sputum, hemoptysis, fever (≥38 **°**C), worsening during clinical examination, and the presence of new signs on chest X-rays. A total of 101 adult BR patients between 18 and 85 years of age who had been previously diagnosed using high-resolution computed tomography (HRCT) of the chest were evaluated. 

**Figure 1:**
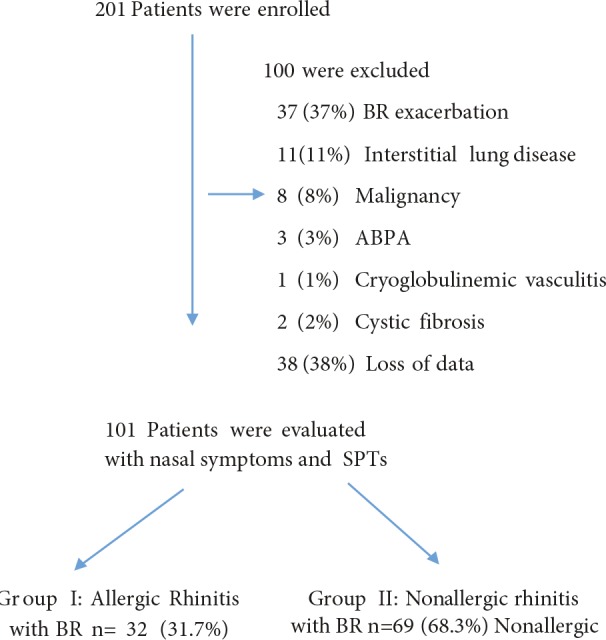
Flowchart of the study. ABPA: Allergic bronchopulmonary aspergillosis, SPT: skin prick test, BR: bronchiectasis.

Demographic (age, sex, and smoking history) and clinical data (respiratory symptoms, nasal symptoms, duration of illness, pulmonary function tests results (forced expiratory volume in 1 s (FEV1), forced vital capacity (FVC), FEV1/FVC), admission to the emergency department (ED) in the last year, and hospitalization in the last year) were recorded. 

The bronchiectasis severity index (BSI) was applied to determine disease severity. The BSI has 8 parameters: age, body mass index (BMI), FEV1 % predicted, hospital admission in the last 2 years, exacerbation frequency in the last year, modified Medical Research Council (mMRC) dyspnea scale, sputum colonization status, and radiological severity (10). These scores classify patients into low-, moderate-, and high-risk groups that identify patients at risk of future mortality, hospitalization, and exacerbation. The bronchiectasis etiology and comorbidity index (BACI) was used to assess comorbidity, which carried independent prognostic value related to exacerbation and *Pseudomonas aeruginosa* infection (11).

Atopy was assessed based on a positive SPT reaction to any of the allergens. SPTs were performed by two trained nurses in the Adult Allergy Laboratory using commercial extracts (ALK or Allergon) of *Dermatophagoides pteronyssinus*, *Dermatophagoides farinea*, *Phleum pratense*, *Artemisia vulgaris*, *Betula*, *Parieteria officinalis*, *Blattella*, grass mix, tree mix, *Olea europaea*,* Aspergillus*,* Cladosporium*,* Penicillium*,* Alternaria*, and dog and cat epithelia. Histamine and glycerol solution with phenol were used as positive and negative controls, respectively. Positive skin reaction to an aeroallergen was defined as wheal diameter ≥3 mm compared with the negative control. The test was performed as described in the position paper of the EAACI (12). There were some differences in SPT sensitivity based on the region of Turkey. In our hospital, SPT panels were created based on common aeroallergens in our region by an allergy specialist.

AR was defined by symptoms including nasal blockage, anterior or posterior rhinorrhea, sneezing, and/or itching that occurred during 2 or more consecutive days for more than 1 h on most days and skin test positivity to common aeroallergens (13). We excluded patients who had previously been diagnosed by a doctor with chronic rhinosinusitis, nasal polyposis, and septal deviation. We also excluded those who had all 4 of the cardinal symptoms of chronic rhinosinusitis, including anterior and/or posterior nasal mucopurulent drainage; nasal obstruction/nasal blockage/congestion; facial pain, pressure, and/or fullness; and reduction or loss of sense of smell (13,14).

The study was approved by the local ethics and clinical research committee of our hospital (2016/40). All participants provided written informed consent.

### 2.1. Statistical analysis

Statistical analyses were performed using StataCorp 2015 (Stata Statistical Software: Release 15.1; StataCorp LP, College Station, TX, USA). Descriptive data were presented as the mean and SD or as median (range) when a normal distribution was not identified. Chi-square testing and t-tests were used for categorical and continuous factors, respectively. Correlation of AR with number of admissions to the ED in the previous year was investigated by Spearman correlation test. Statistical significance was considered as P < 0.05. 

## 3. Results

Among 201 patients, 101 were considered eligible for screening (Figure). The mean age of the enrolled patients was 48 ± 15 years; 55 (54.5%) patients were women. The baseline characteristics of the 101 patients are presented in Table 1. The most common comorbidities included gastroesophageal reflux (GOR) (n = 24, 23.8%), chronic obstructive lung diseases (COPD) (n = 18, 17.6%), hypertension (n = 17, 16.8%), asthma (n = 10, 9.8%), coronary artery diseases (n = 5, 4.9%), and diabetes mellitus (n = 4, 3.9%). In the SPT-positive group, comorbidity was less frequent compared with the SPT-negative group (21.6% vs. 42.2%, P < 0.03). Furthermore, no relationship was observed between comorbidities and AR in BR patients (P > 0.05).

**Table 1:** Demographic and clinical data of bronchiectasis patients with or without allergic rhinitis.

Items	All BR patients	BR patients with AR	BR patients without AR	P
Subjects, n (%)	101	32 (31.7)	69 (68.3)	
Sex, female/male, n	55 / 46	16 / 16	39 / 30	0.51
Age (years) Age, years±SD	48.9 ± 15	45.8 ± 2.6	50.4 ± 1.8	0.17
Duration of diagnosis (years) years±SD	7.1 ± 6.0	7.3 ± 5.4	6.8 ± 6.5	0.37
Smoking history n (%)Current smokerEx-smokerNonsmoker	25 (24.8)20 (19.8)56 (55.4)	8 (25)7 (21.8)17 (53.3)	17 (24.6)13 (18.8)39 (56.5)	0.82
Body mass index mean±SD	26.7 ± 4.9	26.5 ± 5.4	26.7 ± 4.7	0.87
BSI*, mean ± SDMild BSI, n (%)Moderate BSI, n (%)Severe BSI, n (%)	3.8 ± 2.767 (66.3)27 (26.7)7 (6.9)	3.6 ± 2.820 (19.8)10 (9.9)2 (2.0)	4.1 ± 2.747 (46.5)17 (16.8)5 (5.0)	0.48
BACIt mean±SD	3.9 ± 2.9	3.8 ± 1.8	3.9 ± 3.1	0.76
Cough n (%)	77 (76.2)	52 (67.5)	25 (32.5)	0.61
Dyspnea n (%)	85 (84.2)	56 (65.9)	29 (34.1)	0.04
Sputum n (%)	57 (56.4)	19 (32.8)	39 (67.2)	0.56
Hemoptysis n (%)	24 (23.8)	11 (18.8)	13 (34.4)	0.19
Nasal blockage n (%)	51 (50.5)	27 (84.4)	24 (34.7)	0.001
Anterior rhinorrhea n (%)	35 (34.7)	18 (53.1)	17 (26.1)	0.01
Posterior rhinorrhea n (%)	42 (41.6)	16 (50)	26 (37.8)	0.18
Sneezing n (%)	28 (27.7)	15 (46.9)	13 (18.8)	0.008
Nasal itching n (%)	19 (18.8)	8 (25.0)	11 (15.9)	0.27
Loss of smell n (%)	29 (34.5)	18 (56.2)	11 (21.1)	0.002

*: Bronchiectasis severity index, etiology, and comorbidity index. t: Bronchiectasis etiology and comorbidity index.

SPT positivity was detected in 37 (36.6%) cases. The most common positive allergens in SPTs were house dust mites (*Dermatophagoides pteronyssinus*, 30 (29.7%) and *Dermatophagoides farinea*, 26 (26.9%)). The other positive aeroallergens included grass pollen (4, 3.9%) and *Blattella* (4, 3.9%). Polysensitization to aeroallergens, defined as more than one kind of allergen sensitization, was found in 29 (78.4%) of SPT-positive patients. The most common polysensitization aeroallergens were *Dermatophagoides pteronyssinus* and *Dermatophagoides farinea*.

AR was detected in 32 (31.7%) patients with BR. Patients were divided into two groups according to the prevalence of AR. Demographic and clinical characteristics of the groups are presented in Table 1. No statistically significant differences were observed between the groups regarding sex, age, smoking history, BMI, BSI, or respiratory symptoms such as cough, sputum production, and hemoptysis (Table 1). AR was related to dyspnea, nasal blockage, anterior rhinorrhea, sneezing, loss of smell, and number of admissions to the ED in the previous year (Table 1). The mean FEV1 and FVC values were 1.8 ± 0.9 L and 2.4 ± 0.9 L, respectively. BR patients with AR had higher FEV1 and FVC values than the other group (Table 2). Although there was no correlation between AR and admission to the ER in the previous year (r = 0.088, P < 0.05), the number of emergency room visits was higher in the AR group (r = 0.417, P = 0.005). There were also no statistically significant differences between seasons and SPT positivity (P = 0.347).

**Table 2:** Clinical data of bronchiectasis patients with or without allergic rhinitis.

Items	All BR patients,n = 101	BR patientswith AR, n = 32	BR patientswithout AR, n = 69	P
Skin prick test positivity, n (%)	37 (36.6)	32 (31.6)	5 (4.9)	0.001
FEV1*, L	1.8 ± 0.9	2.1 ± 1.1	1.7 ± 0.7	0.01
FVCt, L	2.4 ± 0.9	2.8 ± 1.1	2.3 ± 0.8	0.01
Blood eosinophil/µL	234 ± 164	252 ± 173	224 ± 160	0.58
Drug hypersensitivity	6 (5.9)	3 (2.9)	3 (2.9)	0.91
Admission to EDŧ in previous year	43 (42.6)	28 (65.1)	15 (34.9)	0.09
Number of admissions to ED in previous year	2.7 ± 1.8	3.2 ± 1.6	1.9 ± 1.8	0.01
Hospitalization	9 (8.9)	3 (9.4)	6 (8.7)	0.73

Data presented as mean (standard deviation) or number (%). *: Forced expiratory in 1 s, t: forced vital capacity, ŧ: emergency department.

Self-reported drug hypersensitivity (DHS) was observed in 6 (5.9%) patients, 4 of whom had nonsteroidal antiinflammatory drug hypersensitivity and 2 of whom had hypersensitivity to penicillium-group antibiotics; no differences were noted between the two groups. In the SPT-positive group, comorbidity was less frequent compared with the SPT-negative group (21.6% vs. 42.2%, P < 0.03). In addition, this difference was not detected between the AR groups. However, in the SPT-positive group, sneezing was more frequent compared with the SPT-negative group (40.5% vs. 20.3%, P = 0.03). 

The mean BSI score was 3.8 ± 2.7, and patients were classified into mild (BSI score 0–4), moderate (BSI score 5–8), and severe (BSI score 9+) disease categories. No statistically significant difference was observed between BSI category and AR/SPT positivity.

## 4. Discussion 

In the present study, we showed that the prevalence of AR was 31.7% in adult non-CF BR patients. We also found that atopy was detected in more than one-third of adult BR patients. Presence of AR in adult BR patients was associated with increased dyspnea and number of admissions to the ER in the previous year.

Limited studies have investigated atopy in BR. Most of these were published in 2006 (15–18). Ozturk et al. studied the relationship between atopy and radiologic extent on HRCT and PFTs in 121 adult BR patients. In total, 48.8% of patients were atopic. Given that the study was conducted in a military hospital, most of the patients were male (93.4%) and young (24.6 ± 6.7 years of age) (18). In our study, atopy was detected in 36.6% of adult patients with BR, and the study population was homogeneous in sex. The mean age of our study population was older than Ozturk et al.’s study population. Pang et al. evaluated atopy and bronchial hyperreactivity in BR and control patients. Atopy was reported in 9/36 (25%) of the BR group and 5/36 (13.8%) of the control group. However, no statistically significant difference was found for bronchial hyperreactivity between the 2 groups (15). In 1984, Murphy et al. investigated atopy in 23 BR patients diagnosed bronchographically. In total, 30.4% of BR patients were atopic (16). Except for Ozturk et al.’s research, most of the previous studies used different methodologies from our study, which used bronchography for the diagnosis of BR and had small sample sizes (5,15,16). In recent Turkish studies, the atopy rate was reported as 14.6%–25% in the general population (18,19). In our results, the atopy rate was higher compared with rates of other studies. In the etiopathogenesis of asthma, the presence and importance of atopy has been demonstrated in various studies (20). In addition, BR was detected by thorax CT in 24.8% of asthma patients (21). In our study group, only 9% of the adult BR patients had asthma. Therefore, detection of atopy may be important in the etiopathogenesis of BR without asthma. Further research should be performed in this regard.

Recent studies evaluated sinonasal diseases and BR (9,22,23). In the study of Guilemany et al., a high prevalence of chronic rhinosinusitis (CRS) (77%) and nasal polyposis (NP) (26%) related to the severity of BR was noted. BR patients with CRS and NP exhibited more active diseases and extended airway inflammation (9). Another study demonstrated that sinonasal diseases negatively affected the quality of life in BR cases (8). However, studies on the presence of allergic rhinitis and the clinical effects in adult BR cases are lacking. In our study, the frequency of AR was 31.6%. In the English-language medical literature, the prevalence of AR has been reported as 12.7%–28% in the general population (19,23). The Prevalence and Risk Factors of Allergy in Turkey (PARFAIT) study evaluated the prevalence of allergy in adults. The researchers reported that the prevalence of AR was 17.2% (24). They also showed that females had more AR than males. In our study, the prevalence of AR with adult BR was higher than in other studies, without differences by sex.

In our study, there was no relation between BR and AR admission to the ED in the previous year. However, it was reported that BR patients with AR had an increased number of admissions to the ED in the previous year. To our knowledge, there has not been any previous study that investigated adult BR patients with AR in relation to admission to the ED. In Brandao et al.’s study, patients with asthma and AR using nasal steroids were not hospitalized, and the frequency of ER visits was lower (25). Some studies reported that treatment of AR might result in improved asthma control and reduced risk of emergency room visits and hospitalization in patients with asthma and AR (26,27). Although it might be an emergency referral for BR, we think that nasal congestion, which is the cardinal symptom of AR, could increase the perception of dyspnea, thus affecting the number of admissions to the ED. However, this result should be checked with a large number of BR patients.

Aliberti et al. defined 4 phenotypes of BR: 1) presence of chronic infection with *Pseudomonas aeruginosa*; 2) presence of chronic infection without *Pseudomonas aeruginosa*; 3) daily sputum; and 4) dry bronchiectasis (28). To our knowledge, atopic bronchiectasis has not been previously defined as a phenotype in the English-language literature. In the presence or absence of asthma, atopy (and allergic rhinitis) may represent a new phenotype in BR cases. Our hypothesis should be addressed in future studies.

Yang et al. reported that BR was more common and more prevalent in COPD patients with CRS. It was demonstrated that CRS was related to impaired pulmonary function tests and increased respiratory symptoms, such as dyspnea and cough (29). It was also reported that noninfectious rhinitis was increased in COPD patients, which was related to smoking and atopy. The presence of noninfectious rhinitis with COPD might be associated with both upper and lower respiratory tract inflammation (30). In our study, AR detection in BR cases was found to be related to increased dyspnea. We thought that this might be associated with nasal obstruction. However, we have not questioned whether the nasal symptoms or respiratory symptoms started earlier.

In our study groups, FEV1 and FVC were lower in the patients with BR without AR. It is difficult to explain these findings, because there were no statistically significant differences between age, sex, smoking history, comorbidity, diagnosis of asthma, BSI, or radiologic disturbances in thorax CT. It might be related to duration of diagnosis, which was longer in BR patients with AR. That means that BR patients with AR might be using inhaled medication, which protects lung function from declining.

Sneezing in the SPT-positive group was significantly more frequent than in the SPT-negative group (40.5% vs. 20.3%). This might be due to the mean age of the SPT-negative patients being higher than that of SPT-positive patients. Additionally, although this difference was not detected in AR patients, in the SPT-positive group, the frequency of sneezing was more frequent than in the negative group.

For all study populations, SPT positivity was detected in 36.6% patients, and AR was found in 31.7% of them. However, 5 patients who were SPT-positive had not been diagnosed with AR or another atopic disease. Since atopic diseases such as AR, asthma, or allergic conjunctivitis may develop in the follow-up for SPT-positive cases, it may be necessary to be careful with patients with BR.

In this study, self-reported DHS was detected in 5.9% adult patients with BR. The majority of these patients had antibiotic hypersensitivity, especially to penicillin and cephalosporin. It has been previously shown that DHS was present in 7%–12% of the general population (31,32). Data on DHS in BR patients are not available. The drug allergy diagnosis was made according to patient self-reports. These patients were evaluated for DHS in an adult allergy unit.

Finally, the most common comorbidity was GOR in all study groups. In the SPT-positive group, comorbidity was less frequent compared with the SPT-negative group (21.6% vs. 42.2%). This difference might be due to the older age of the SPT-negative group. However, there was no significant difference in comorbidities between the 2 groups.

Our study has some limitations. First, the number of patients might not completely represent BR patients in our country. Our hospital is a tertiary chest disease and thoracic surgery hospital, so selective BR patients might be admitted to our outpatient clinics. We excluded patients with chronic rhinosinusitis, nasal polyposis, or septal deviation. Furthermore, we did not classify patients according to those who had persistent or intermittent AR symptoms. As this study was retrospective, we only excluded according to history, symptoms, and signs recorded in the file; we did not refer patients to the rhinology unit for newly diagnosed nasal and paranasal sinus pathologies. 

In conclusion, patients with bronchiectasis are at an increased risk for atopy and AR. Untreated and undetected AR may increase physical disability, morbidity, and health care utilization. Future research is needed to address the impact of the early detection and management of atopic diseases in bronchiectasis.
